# CRISPR mediated targeting of *DUX4* distal regulatory element represses DUX4 target genes dysregulated in Facioscapulohumeral muscular dystrophy

**DOI:** 10.1038/s41598-021-92096-0

**Published:** 2021-06-15

**Authors:** Sunny Das, Brian P. Chadwick

**Affiliations:** 1 Department of Biological Science, Florida State University, Tallahassee, FL 32304 USA; 2 Present Address: Whitehead Institute for Biomedical Research, Cambridge, MA 02142 USA

**Keywords:** Genetic engineering, Reverse transcription polymerase chain reaction, Chromatin, Neuromuscular disease

## Abstract

Facioscapulohumeral muscular dystrophy (FSHD) is a debilitating muscle disease that currently does not have an effective cure or therapy. The abnormal reactivation of *DUX4*, an embryonic gene that is epigenetically silenced in somatic tissues, is causal to FSHD. Disease-specific reactivation of *DUX4* has two common characteristics, the presence of a non-canonical polyadenylation sequence within exon 3 of *DUX4* that stabilizes pathogenic transcripts, and the loss of repressive chromatin modifications at D4Z4, the macrosatellite repeat which encodes *DUX4*. We used CRISPR/Cas9 to silence *DUX4* using two independent approaches. We deleted the *DUX4* pathogenic polyadenylation signal, which resulted in downregulation of pathogenic *DUX4-fl* transcripts. In another approach, we transcriptionally repressed *DUX4* by seeding heterochromatin using the dCas9-KRAB platform within exon 3. These feasibility of targeting *DUX4* experiments were initially tested in a non-myogenic carcinoma cell line that we have previously characterized. Subsequently, in an immortalized patient myoblast cell line, we demonstrated that targeting *DUX4* by either approach led to substantial downregulation of not only pathogenic *DUX4* transcripts, but also a subset of its target genes that are known biomarkers of FSHD. These findings offer proof-of-concept of the effect of silencing the polyadenylation sequence on pathogenic *DUX4* expression.

## Introduction

FSHD is the third most common form of muscular dystrophy, affecting about 1 in 15,000 live births^1^. An autosomal dominant disease, adult-onset FSHD consists of appearance of symptoms in the late twenties or thirties, with subsequent progressive degeneration of muscles of the face, shoulder blades, and upper arms^2,3^. With roughly one-fifth of patients being confined to a wheelchair by age 50, this is an extremely debilitating condition^4^ involving expensive palliative care, and currently does not have a cure or effective therapy.

Two known forms of this disease (FSHD1 & FSHD2; OMIM 158900 and 158901, respectively) are both caused by the activation of a germline-specific gene *DUX4*^5,6^, coded by each tandem repeat unit of a macrosatellite on chromosome 4q35.2 known as D4Z4^7,8^. DUX4 is an important developmental transcription factor that is normally active during a specific period in cleavage stage embryos, activating target genes that drive the embryo towards totipotency^9–11^. However, this gene is epigenetically silenced in somatic cells due to enrichment of repressive modifications, primarily histone H3 tri-methylated at lysine 9 (H3K9me3) and CpG methylation^12–14^. Both forms of FSHD are characterized by chromatin de-repression due to loss of H3K9me3 at D4Z4, on a disease-permissive (4qA) chromosomal background^14,15^. A permissive 4qA haplotype is characterized by presence of multiple single nucleotide polymorphisms (SNPs), the most important of which, creates an additional, non-canonical polyadenylation (poly-A) signal (ATTAAA) in exon 3 of *DUX4*^5^. While each repeat of D4Z4 harbors two exons (Exons 1 and 2) of DUX4, Exon 3 is single copy and resides immediately downstream of the most distal D4Z4 repeat. While regular 4qA transcripts arising in the germline may be stabilized by a distal poly-A, downstream of *DUX4* exon 7^16^, pathogenic transcripts (*DUX4-fl*) arising from somatic cells due to de-repression, utilize the non-canonical exon 3 poly-A^5,17^. Experimental overexpression of DUX4 protein in human myoblasts dysregulates a downstream cascade of genes and muscle-specific regulatory regions that have been implied to play a role in muscle atrophy, inhibition of muscle replenishment and inflammatory immune response^18^.

Given the strong association of FSHD with the presence of DUX4 exon 3 poly-A and heterochromatin loss, we wanted to target this region using two independent approaches with one common goal, downregulation of pathogenic *DUX4-fl*. In the first approach, our aim was to delete the endogenous pathogenic poly-A, while in an independent second approach, we wanted to induce heterochromatin enrichment at exon 3, using the CRISPR platform. To demonstrate the feasibility of targeting a heterochromatic region such as D4Z4, we carried out our initial targeting experiments in HCT116, a carcinoma cell line that we have recently shown to harbor a disease permissive exon 3 poly-A signal^19^, and a DNA methyltransferase knockout (DKO) of HCT116. These cells lines are amenable to gene targeting^20,21^ and are poised for expression of pathogenic *DUX4-fl* transcripts^19^.

To further examine the effect of such targeting on DUX4 target genes in clinically relevant cell types, we carried out subsequent experiments in an hTERT-immortalized FSHD myoblast cell line^22^ with the aim of validating our initial findings in a clinically relevant setting.

## Results

### CRISPR/Cas9 mediated targeting of DUX4 exon 3 poly-A using a paired sgRNAs in HCT116 and DKO cells

**Figure 1:**
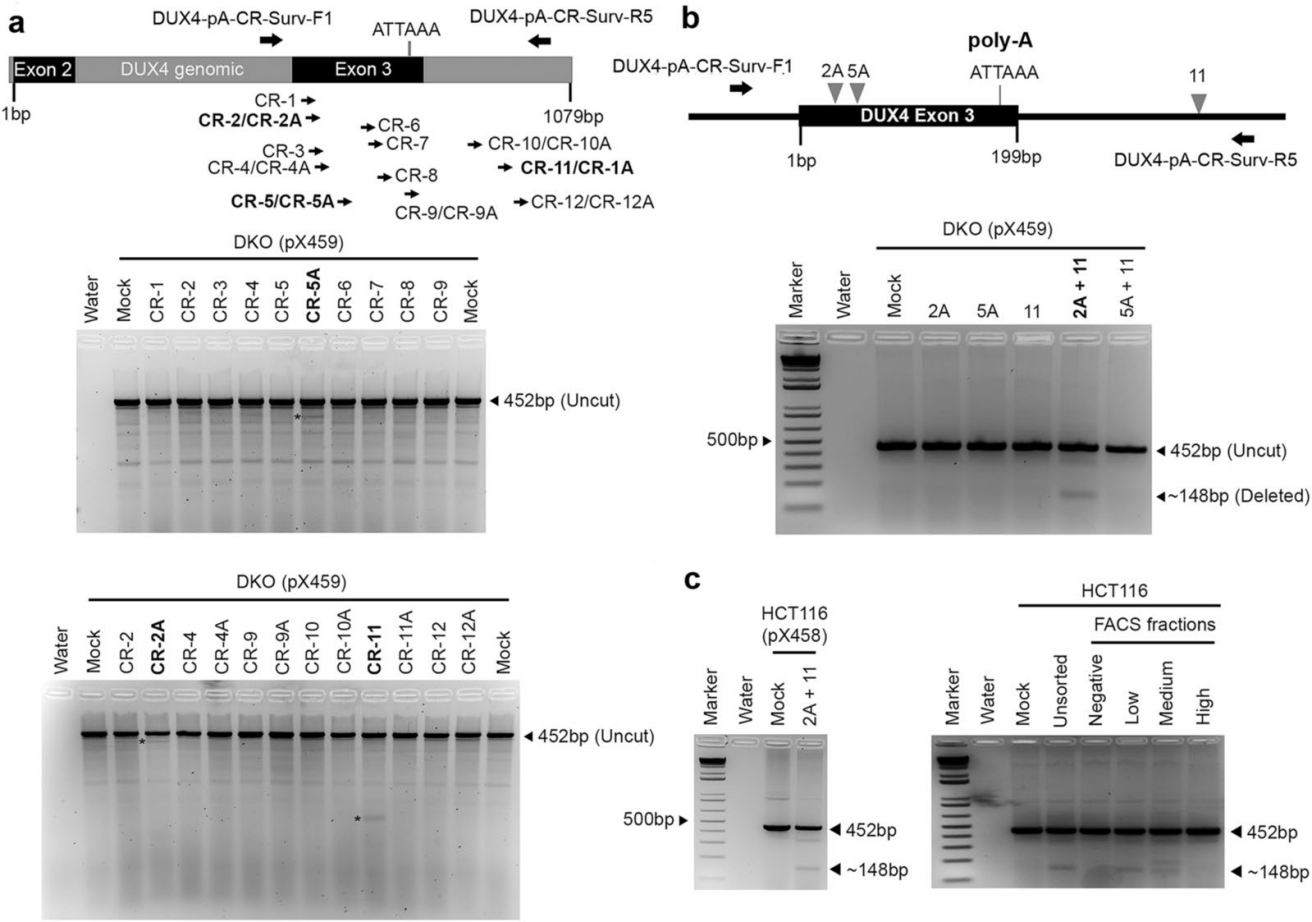
Identification of suitable sgRNAs to target DUX4 poly-A signal in HCT116 and DKO. (**a**) Top panel: Schematic of binding sites for sgRNAs (CR-1 to CR-12) across DUX4 exon 3 and a region downstream of it, which flank and span the poly-A site. sgRNAs with suffix ‘A’ are truncated (17–18 bp) versions of regular sgRNAs (20 bp) with same numerical value. The position of primers used for Surveyor detection of cut and uncut products are shown as black arrows, along with their names. The position of the pathogenic poly-A signal is also indicated. Arrows for efficient sgRNAs are highlighted in bold. The base pair length is indicated to provide a scale. Bottom panels: Ethidium bromide stained agarose gels depicting results of Surveyor assay for these sgRNAs. Sample names are indicated on top along with cell line used for transfection and vector in which sgRNAs were cloned. The expected size for uncut PCR product is indicated on the right-hand side by a black triangle. Efficient sgRNAs are highlighted in bold and the bands representative of their cutting is indicated by an asterisk on the left of the lane corresponding to a given sgRNA (**b**) Top panel shows schematic of trial for a dual-sgRNA PCR based deletion assay with suitable combinations sgRNAs upstream (CR-2A or CR-5A) and downstream (CR-11) of the poly-A site. The position of primers used for PCR detection of cut and uncut products are shown as black arrows, along with their names. Bottom panel shows results of deletion assay using abovementioned sgRNAs. Sample names are indicated on top along with cell line used for transfection and vector in which sgRNAs were cloned. The expected size for undeleted and deleted PCR products is indicated on the right-hand side by black triangles. The most efficient sgRNA combination is highlighted in bold. (**c**) Left panel shows results of a trial deletion assay using abovementioned sgRNAs cloned in pX458 and transfected in parental HCT116 cells. Bottom right panel shows results of deletion assay using abovementioned sgRNAs cloned in pX458 in HCT116 cells harvested before (Unsorted) or after (Negative, Low, Medium and High fluorescence intensity above background) FACS based sorting for GFP-positive cells. Sample names are indicated on top. Schematic diagrams were generated using Microsoft PowerPoint 16.48 (https://www.microsoft.com) and Adobe Photoshop 21.0.1 (https://www.adobe.com).

Given that the exon 3 non-canonical poly-A sequence is strongly associated with pathogenicity of DUX4, we wanted to first assess the feasibility of disruption of this sequence. We chose HCT116, a colon carcinoma cell line and its double-knockout derivative (DKO) that has both DNA methyltransferase genes, *DNMT1* and *DNMT3B* knocked out^21^, for these initial targeting experiments, based on our previous study wherein we demonstrated the presence of the exon 3 pathogenic poly-A in these cell lines^19^. In that study, we had also shown that pathogenic *DUX4* mRNA is transcribed readily in DKO but not in HCT116, owing to repressive chromatin modifications at D4Z4 in the latter. We initially designed and tested multiple single-guide RNAs (sgRNAs) around *DUX4* exon 3 poly-A sequence (Fig. 1a; schema) for their binding and cutting efficiency, using an agarose-gel based surveyor assay^23^ to detect insertions and deletions (indels) generated though the imprecise non homologous end joining double strand break repair pathway. Although no single sgRNA was able to direct Cas9 to cut precisely within the ‘ATTAAA’ sequence (CR-9 encompasses the polyA; Fig. 1a; top gel), we identified three efficient sgRNAs (Fig. 1a; top and bottom gels) that were used for downstream experiments. Other sgRNAs (CR-2 and CR-4) showed similar banding patterns as CR-2A but we chose the latter for downstream experiments, since the signal intensity from this sgRNA was the strongest. Since we had sgRNAs both upstream (CR-2A and CR-5A) and downstream (CR-11) of the poly-A sequence, we tested them in combinations initially in the DKO cell line, to see if they deleted out the intervening region. sgRNAs were cloned in the pX459 (Puromycin) vector to facilitate future isolation of single cell clones using puromycin selection. We designed a PCR based deletion assay for this combination of two target-flanking sgRNAs (Fig. 1b; schema) that allowed us to amplify across the region in both deleted and undeleted genomic sequences. Only one of the two combinations (CR-2A with CR-11) was successful in deleting the desired region (Fig. 1b; gel). However, subsequent attempts to isolate single-cell clones of poly-A deleted DKO cells did not yield any results. This may be attributed to the poor growth of DKO single cells in culture. Owing to difficulties associated with puromycin-selection based single-cell cloning with slow growing DKO cells, namely that any clone isolated would constitutively express the gRNA and Cas9 nuclease and potentially cause problems, we subsequently cloned the sgRNAs into the pX458 vector (same backbone as pX459) that facilitated fluorescence-activated cell sorting (FACS) based enrichment for positively targeted cells using GFP fluorescence above background (Supplementary Fig. S1 online), but we once again failed to isolate single cell clones of targeted DKO cells by this method.

**Figure 2:**
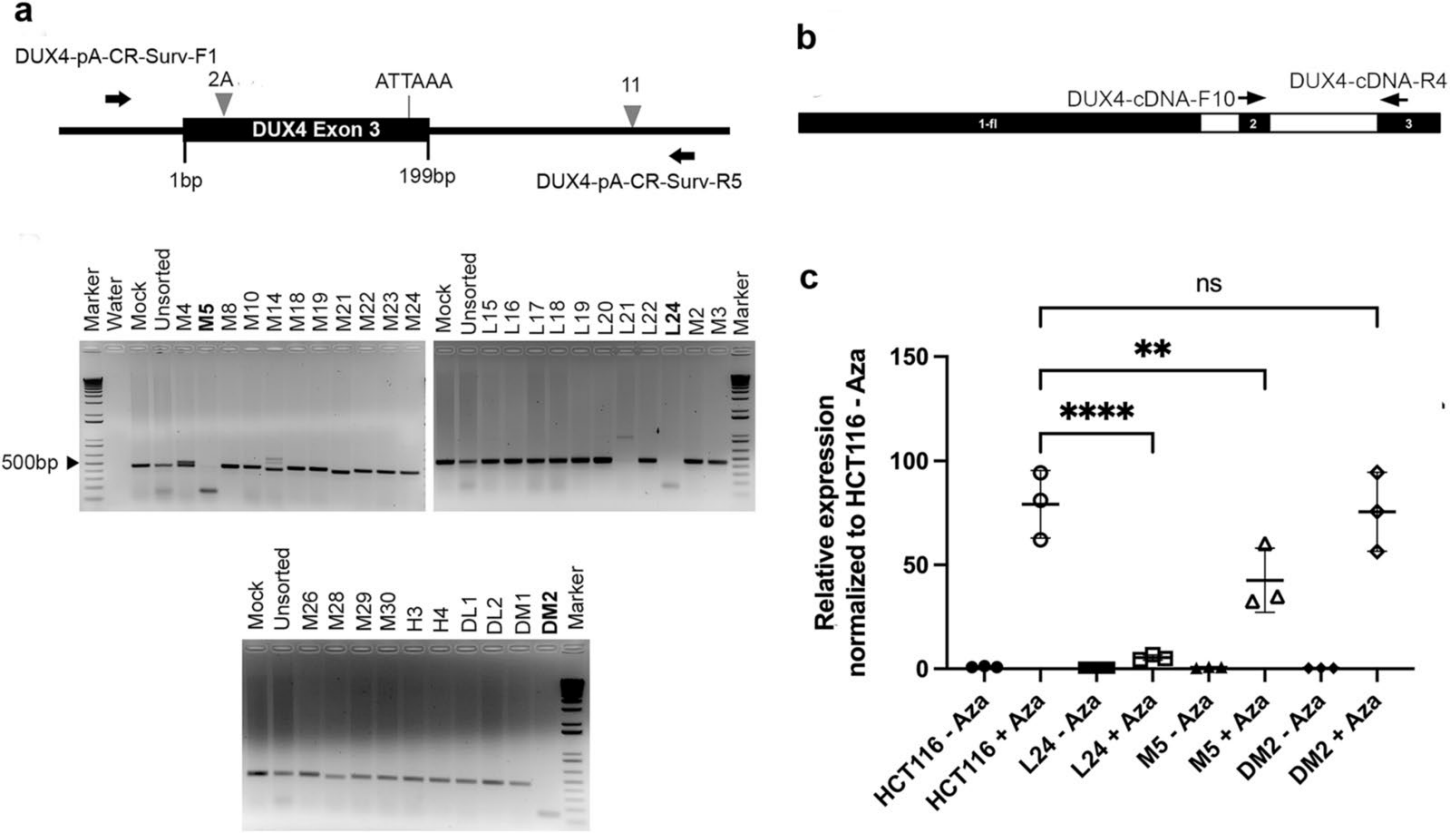
Isolation of DUX4 poly-A targeted single cell clones in HCT116 and transcription of DUX4 and target genes in targeted clones. (**a**) Top panel shows schematic of the dual-sgRNA PCR based deletion assay with the most suitable combination of sgRNAs upstream (CR-2A) and downstream (CR-11) of the poly-A site. The position of primers used for PCR detection of cut and uncut products are shown as black arrows, along with their names. Bottom panels show representative ethidium bromide stained agarose gels showing deletion assay results in single cell clones of HCT116 targeted with CR-2A and CR-11 sgRNAs. Sample names are included on top along with clone numbers assayed. Clones that were subsequently sequenced and analyzed are highlighted in bold. (**b**) Labeled arrows show location of forward and reverse primers, relative to the most distal D4Z4 monomer for detection of spliced and polyadenylated *DUX4-fl* transcripts by qRT-PCR with cDNA samples made with oligo-dT primers. (**c**) Scatterplots showing results from qRT-PCR analysis for *DUX4-fl* in HCT116 and three independent DUX4 poly-A knockouts (named on the X-axis) with or without 5-Aza-C treatment. Transcript levels (Y-axis) are expressed as fold change normalized to expression level in untreated parental HCT116 cells, relative to GAPDH expression. All values are obtained by averaging results from triplicates for each sample. Error bars represent standard deviation (n = 3). Statistical significance is indicated (**p ≤ 0.01, ****p ≤ 0.0001, *ns* no significant difference; *p* > 0.05). Schematic diagrams were generated using Microsoft PowerPoint 16.48 (https://www.microsoft.com) and Adobe Photoshop 21.0.1 (https://www.adobe.com).

Given that we have had success in isolating single cell clones of targeted HCT116 cells in unrelated experiments, we instead used the parental HCT116 cell line to carry out these targeting experiments. Using the CR-2A and CR-11 sgRNAs cloned in pX458 (GFP), we first validated their activity in HCT116 cells (Fig. 1c; left gel). Next, we performed the deletion assay in GFP-enriched fractions (Fig. 1c; right gel) that had been sorted according to different GFP-fluorescence intensities above background, to avoid GFP-mediated cytotoxicity as has been reported in the literature^24,25^ (See “Materials and methods” section). Subsequently, we grew single-cell derived clones from these fractions, screened 60 such clones using the deletion assay (Fig. 2a), and initially sequenced deletion PCR products for six clones (Table 1) that had a putative deletion in the desired region.

**Table 1:** Summary of sequencing results in *DUX4* exon 3 poly-A targeted single-cell clones of HCT116.

Clone	Best match	Description
L21	99.5% Chr10	–
L24	99.4% Chr4	Single sequence with a 303 bp deletion spanning poly-A
M5	100% Chr4	Dominant sequence shows a 296 bp deletion Secondary sequence shows a 15 bp deletion at CR-11 and intact poly-A
M21	99.3% Chr4	Deletion of 29 bp at CR-11 Intact poly-A sequence
M28	99.6% Chr4	Deletion of 17 bp at CR-11 Intact poly-A sequence
DM2	100% Chr4	Dominant sequence shows a 303 bp deletion spanning poly-A Secondary sequence shows no deletion with an intact poly-A

Based on the sequencing results (Table 1; see Supplementary Fig S2 online), we selected three clones, L24, M5 and DM2 for downstream analysis. In a previous study^19^, we have shown that the HCT116 cell line is negative for the 4qB allele, and based on genotyping experiments in this previous study, we estimate that at least one of its two 4qA alleles is the pathogenic haplotype due to the presence of the ‘ATTAAA’ sequence, and it is very likely that HCT116 has two permissive/pathogenic 4qA haplotypes (such as 4qA161 and 4qA-L)^5^.

It is to be noted that sequencing primers used for deletion assay, DUX4-pA-Surv-F1/R5/R6, can also detect the same region in 10q but the ATTAAA pathogenic poly-A sequence seen in clones with no poly-A deletion (e.g., M21/M28; Table 1) is specific to 4qA, and is absent in 10q. If the sequence is from 10qA, one would expect to see an ‘ATCAAA’ in place of the ATTAAA, as seen in Clone L21 (Table 1), suggesting that the reported sequence indeed arise from pathogenic 4qA.

Next, we tested how the deletion of the exon 3 poly-A affected their potential to produce *DUX4-fl* transcripts. To this end, we treated parental HCT116 cells and the three mutant clones with 5-Aza-deoxycytidine (5-Aza-C), which is a known inhibitor of DNA methyltransferases^26^, and upregulates the transcription of a significant fraction of the genome^20,21^, including the D4Z4 locus in HCT116 cells^19^. Nested RT-PCR has been previously used for detecting extremely low levels of *DUX4* mRNA in patient samples^5,16^. However, given that in our experiments, we were treating cells with 5-Aza-C, which induces high levels of *DUX4* expression, we instead resorted to an indirect method to quantitate how efficient our targeting strategy was, using quantitative reverse transcriptase PCR. We have previously^19^ used a primer pair that anneal to *DUX4* exons 2 and 3 (Supplementary Table S1) to carry out qRT-PCR using oligo-dT primed cDNA. This selectively amplifies polyadenylated and spliced pathogenic *DUX4-fl* arising from splicing of transcripts across exons 2 and 3 from the most distal D4Z4 repeat, and also leaves out amplification of general D4Z4 transcripts arising from other repeats. While the forward qRT-PCR primer used for detecting *DUX4-fl* (DUX4-cDNA-F10) anneals only to chromosome 4q, the reverse primer (DUX4-cDNA-R4) can anneal to both 4qA and the highly homologous 10qA^5^. However, even in the presence of a pLAM sequence and exon 3 on 10qA, transcripts arising from this chromosome are unstable due to a lack of the pathogenic poly-A (it instead has the ‘ATCAAA’ sequence)^5,19^, and consequently our qRT-PCR protocol will not amplify such 10qA transcripts. Thus, our qRT-PCR protocol will selectively amplify spliced and polyadenylated pathogenic *DUX4-fl* arising from a disease-permissive 4qA allele. However, it is important to note that our qRT-PCR reverse primer normally anneals to a region within exon 3, just downstream of the CR-2A sgRNA binding site (Fig. 2b). Thus, any successful deletion of the targeted region between CR-2A and CR-11 will abolish this annealing site for the reverse primer and not result in detection of transcript levels. Stated differently, using this approach, we do not directly quantitate the steady state levels of *DUX4* mRNA after targeting but rather, we are able to indirectly assess the amount of un-targeted *DUX4* in various clones. Based on sequencing results, using this strategy, clone L24 should show low levels of *DUX4-fl* transcript likely due to the inability of the qRT-PCR reverse primer to bind to its annealing sequence, since the entire region has been deleted. Thus, while using this exon 2–3 primer set would validate the deletion, it is insufficient to rule out the inability of the poly-A deleted clone L24 to generate stable *DUX4-fl* transcripts. It should be noted this deleted clone retains some residual levels of DUX4 expression and does not show a total abolishment of expression. The inability to completely abolish *DUX4-fl* expression might arise from some 10q-derived mRNA being made. This possibility cannot be completely ruled out, especially in the context of the harsh 5-Aza-C treatment of targeted cells. In this regard, using primers upstream of exon 3, i.e., within exons 1 or 2 with oligo-dT primed cDNA may ostensibly appear to offer some resolution on this shortcoming. However, the strong demethylation due to 5-Aza-C results in upregulation of not only *DUX4-fl* transcripts but also general D4Z4 transcription in parental HCT116 cells (unpublished results). Thus, it is expected that in similarly treated poly-A deleted clones (such as L24) the use of Exon 1–2 primers will show high D4Z4 transcription. Effectively, this will make it difficult to evaluate if the Exon 3 poly-A targeting strategy worked or not. In contrast, as mentioned previously, the use of Exon 2–3 primers can at least indirectly evaluate the levels of undeleted pathogenic *DUX4-fl* specifically and not general D4Z4 transcription. With this in mind, we carried out qRT-PCRs with the Exon 2–3 set of primers on 5-Aza-C treated parental HCT116 and the three targeted clones. With this primer pair, as expected, we observed significantly low levels of *DUX4-fl* in clone L24 upon treatment, compared to treated controls (HCT116) (Fig. 2c). We only observed a partial reduction in *DUX4-fl* transcript levels in clone M5 and no reduction in Clone DM2. This can likely be explained by the fact that although the sequencing result of the deletion PCR product in these clones show a dominant trace of poly-A deletion (Supplementary Fig S2), we also detect a second weak trace in M5 and DM2. For clone M5, this second trace contains the poly-A sequence and a small deletion at one of the two gRNA sites, whereas in DM2 the second trace contains the poly-A, but no other damage. In contrast, L24 did not show the presence of a second sequence trace. Therefore, we interpret that HCT116 cells possess two 4qA alleles, with at least one as pathogenic. In L24, both alleles appear to be deleted and we detect very low levels of *DUX4-fl*. In M5, one 4qA allele is deleted while the second that carries the poly-A signal is damaged, which may be sufficient to destabilize transcripts as DUX4-fl levels are significantly reduced in this clone. Finally, DM2 shows loss of one 4qA allele, but the second pathogenic allele remains intact potentially explaining why this clone shows DUX4-fl that are comparable to parental Aza-treated cells (Fig. 2c).

We initially attempted to assess how the loss of DUX4 exon 3 poly-A affects the expression of its putative target genes, such as *TRIM43* and *MBD3L2*^27^ using qRT-PCR for these genes in 5-Aza-C treated parental HCT116 cells and Clone L24. However, we found that both genes were already expressed at high levels in parental HCT116 and the 5-Aza-C treatment did not significantly increase their expression, suggesting that in this cell line their expression is DUX4-independent (data not shown). Subsequently, to validate whether targeting the exon 3 poly-A leads to a loss of DUX4 protein activity, we extended our targeting approach to FSHD myoblasts that naturally express *DUX4-fl*, obviating the need for using 5-Aza-C.

### CRISPR/Cas9 mediated targeting DUX4 exon 3 poly-A in hTERT-immortalized patient myoblasts to downregulate DUX4 and its target genes

**Figure 3:**
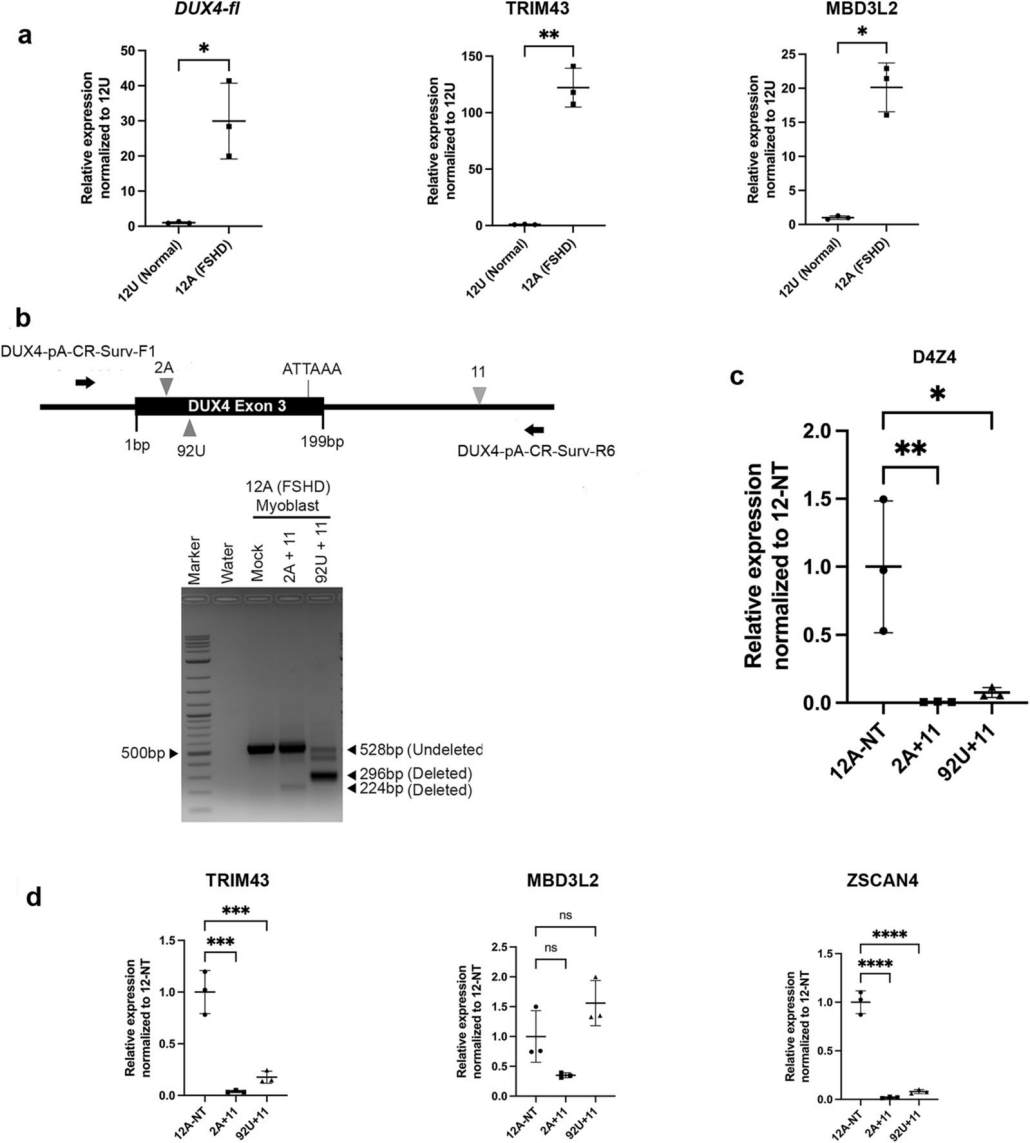
CRISPR-Cas9 mediated targeting of pathogenic poly-A and downregulation of DUX4 and target genes in patient myoblasts. (**a**) qRT-PCR analysis for *DUX4-fl* (left) and target genes *TRIM43* and *MBD3L2* in unaffected and patient hTERT immortalized myoblasts. Transcript levels are expressed as fold change normalized to expression level in unaffected (12U) myoblasts, relative to GAPDH expression. All values are obtained by averaging results from triplicates for each sample. Statistical significance is indicated (*p ≤ 0.05; **p ≤ 0.01). (**b**) Top panel shows schematic of for the dual-sgRNA PCR based deletion assay with two suitable combination of sgRNAs upstream (CR-2A or CR-92U) and downstream (CR-11) of the poly-A site, indicated by triangles. CR-92U binds to the antisense strand, as indicated by inverted triangle. The position of primers used for PCR detection of cut and uncut products are shown as black arrows, along with their names. Bottom panel shows results of a trial deletion assay using abovementioned sgRNAs cloned in suitable lentiviral vectors, transduced in patient myoblast cells. Sample names are indicated on top. The expected size for undeleted and deleted PCR products is indicated on the right-hand side by black triangles. (**c**) qRT-PCR analysis for *D4Z4* in patient (12A) control (NT) myoblasts, along with cells treated with different sgRNA combinations (named on the X-axis) targeting DUX4 poly-A. Transcript levels (Y-axis) are expressed as fold change normalized to expression level in control (NT) myoblasts, relative to ACTB expression. All values are obtained by averaging results from triplicates for each sample. Error bars represent standard deviation (n = 3). Statistical significance is indicated (*p ≤ 0.05; **p ≤ 0.01). (**d**) qRT-PCR analysis for DUX4 target gene expression in patient myoblast controls and cells treated with different sgRNA combinations (named on the X-axis) targeting DUX4 poly-A. Transcript levels (Y-axis) for *TRIM43*, *MBD3L2* and *ZSCAN4* are expressed as fold change normalized to expression level in control (NT) patient myoblasts, relative to ACTB expression. All values are obtained by averaging results from triplicates for each sample. Statistical significance is indicated (***p ≤ 0.001; ****p ≤ 0.0001; *ns* no significant difference; *p* > 0.05). Schematic diagrams were generated using Microsoft PowerPoint 16.48 (https://www.microsoft.com) and Adobe Photoshop 21.0.1 (https://www.adobe.com).

We first characterized two hTERT-immortalized myoblast cell lines derived from the deltoid muscles of a female FSHD patient (12A) and her unaffected sibling (12U), by assessing expression levels of *DUX4-fl* and target genes *TRIM43* and *MBD3L2*. As expected, we found significantly high levels of *DUX4* and target gene expression in the patient myoblasts as compared to the unaffected control (Fig. 3a; see Supplementary Table S2 online).

Due to poor transfection efficiency, we carried out lentiviral transduction of our sgRNAs and Cas9 in the hTERT-immortalized FSHD myoblast cell line (12A). Initially, we adapted publicly available protocols (https://portals.broadinstitute.org/gpp/public/resources/protocols), and developed a protocol that consistently generates high titers of viral particles and we were also able to infect myoblasts with high (90–100%) efficiency (See “Materials and methods” section; data not shown). We cloned CR-2A and CR-11 in suitable lentiviral vectors and transduced 12A myoblasts (See “Materials and methods” section). During our transition from working with HCT116 to myoblasts, we identified an additional sgRNA (CR-92U; not identified in our initial screening shown in Fig. 1a) upstream of the poly-A, which we found can delete the same intervening region encompassing the poly-A, in combination with CR-11 (Fig. 3b; top panel). Since CR-92U + CR-11 combination was also very efficient in our deletion assay, we included this second pair along with CR-2A + CR-11 to demonstrate the feasibility of targeting this region robustly across multiple sgRNA pairs. Independent transductions of these two sgRNA pairs in myoblasts, followed by deletion assay showed that both pairs can delete the intervening region (Fig. 3b; gel), with the CR-92U/CR-11 pair generating much more deleted product than the CR-2A/CR-11 pair, suggesting that CR-92U might be more efficient in cutting the genomic locus than CR-2A. To this end, we TA cloned and sequenced the PCR products across the target region when using either the 2A-11 or 92U-11 combinations in FSHD myoblasts and the results (Supplementary Table S3A,B) suggest that CR-92U does seem to introduce damage at its cut site more often than 2A.

Since most of the myoblasts had received both sgRNAs (as seen by co-existence of GFP and tRFP signals contained on the two gRNA expression constructs), and since close to 100% of cells were infected, we did not sort cells any further, and proceeded to downstream analyses in pooled transduced myoblasts. We assessed *DUX4-fl* expression in patient (12A) myoblasts transduced with the two sgRNA pairs and compared transcript levels to patient myoblasts transduced with a non-targeting control vector, cloned with a scrambled sgRNA that has no specificity for the target sequence or any other sequence in the human genome. Due to the same caveat we encountered with qRT-PCR in HCT116 cells (inability of exon 3 reverse primer to bind within the deleted region), here we carried out qRT-PCR for D4Z4 transcripts only using exon 1 primers. We observed a significant decrease in D4Z4 transcript levels in poly-A targeted patient myoblasts with both sgRNA pairs, compared to controls (Fig. 3c).

To assess if poly-A deletion had any effect on DUX4 and consequently its downstream targets, using qRT-PCR, we quantified transcript expression levels for three genes, *TRIM43, MBD3L2*, and *ZSCAN4* (Fig. 3d), which are also bonafide biomarkers of FSHD^18^. *ZSCAN4* has recently been shown to be a direct target of DUX4 protein that is activated during the early stages of embryonic development and is partially responsible for fulfilling DUX4’s role as a major player in embryonic gene activation^9^. We found and highly significant downregulation of *TRIM43* and *ZSCAN4* with the CR-2A/CR-11 pair, whereas despite a substantial reduction, change in MBD3L2 levels did not reach significance. In contrast to expectations based on our deletion assay results, there was lesser but significant downregulation with the CR-92U/CR-11 pair for D4Z4 transcripts, *TRIM43* and *ZSCAN4*, whereas there was no significant change with this pair for *MBD3L2*.

### dCas9-KRAB mediated enrichment of H3K9me3 in hTERT-immortalized patient myoblasts to downregulate DUX4 and its target genes

**Figure 4:**
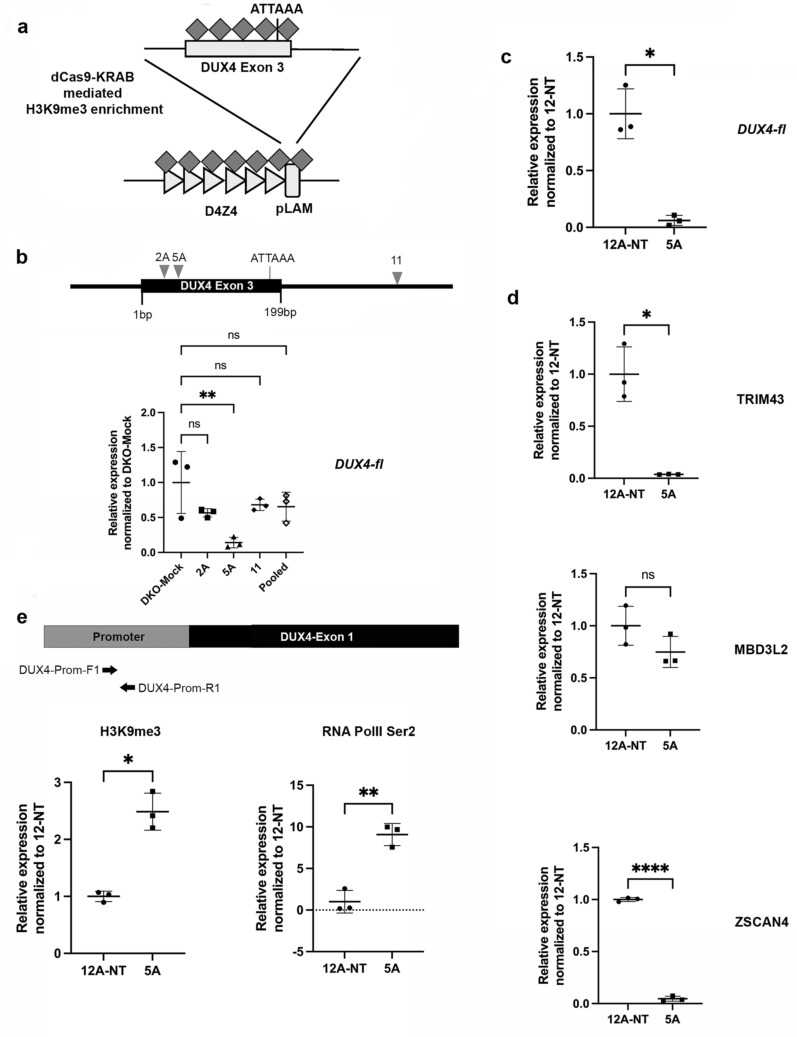
Downregulation of DUX4 and target genes by dCas9-KRAB mediated enrichment of H3K9me3 in patient myoblasts. (**a**) Schematic showing targeting strategy to enrich H3K9me3 and repress DUX4 using a dCas9-KRAB effector recruited by suitable sgRNAs designed at DUX4 exon 3. (**b**) Top panel shows schematic for binding sites of three previously used sgRNAs in DUX4 exon 3. Bottom panel shows qRT-PCR analysis for *DUX4-fl* in mock (dCas9-KRAB only) and sgRNA-transfected DKO cells. Sample names are indicated on the X-axis. sgRNAs were tested individually as well as a pool. Transcript levels (Y-axis) are expressed as fold change normalized to expression level in mock DKO cells, relative to GAPDH expression. All values are obtained by averaging results from triplicates for each sample. Statistical significance is indicated (**p ≤ 0.01; *ns* no significant difference; *p* > 0.05) (**c**) qRT-PCR analysis for *DUX4-fl* in patient myoblast controls, along with cells treated with the sgRNA (CR-5A) Sample names are indicated on the X-axis. Transcript levels (Y-axis) are expressed as fold change normalized to control (NT) patient myoblasts, relative to ACTB expression. All values are obtained by averaging results from triplicates for each sample. Error bars represent standard deviation (n = 3). Statistical significance is indicated (*p ≤ 0.05). (**d**) qRT-PCR analysis for DUX4 target genes *TRIM43*, *MBD3L2* and *ZSCAN4* control and sgRNA (CR-5A) targeted patient myoblasts. Transcript levels are expressed as fold-change normalized to expression level in control (NT) patient myoblasts, relative to ACTB expression. All values are obtained by averaging results from triplicates for each sample. Error bars represent standard deviation (n = 3). Statistical significance is indicated (*p ≤ 0.05, ****p ≤ 0.0001, *ns* no significant difference; *p* > 0.05). (**e**) Results of qPCR for H3K9me3 and RNAPolII-S2 ChIP in control and sgRNA targeted patient myoblasts. Labeled arrows show location of primers in the DUX4 promoter region (grey), relative to the DUX4 ORF (black) within each D4Z4 monomer. Sample names are indicated on the X-axis while enrichment values on the Y-axis are expressed as percentage of corresponding input samples, after normalization with respect to corresponding RS samples. All values are obtained by averaging results from triplicates for each sample. Error bars represent standard deviation (n = 3). Statistical significance is indicated (*p ≤ 0.05, **p ≤ 0.01). Schematic diagrams were generated using Microsoft PowerPoint 16.48 (https://www.microsoft.com) and Adobe Photoshop 21.0.1 (https://www.adobe.com).

Since expression of pathogenic DUX4 in patients is caused due to loss of H3K9me3 mediated heterochromatin, we wanted to see if enrichment of H3K9me3 at D4Z4 would repress *DUX4*. Repressor domains such as Krüppel associated box (KRAB) are found in zinc-finger protein-based transcription factors^28^. A KRAB domain, when tagged with a catalytically inactive ‘dead’ Cas9 (dCas9), can potentially recruit an adapter protein called Tripartite motif containing 28 (TRIM28) to a desired genomic locus. TRIM28 guides SETDB1 to lay down H3K9me3 at the given region (Fig. 4a), which can lead to transcriptional silencing at the desired locus. Using suitable sgRNAs, this strategy has been shown to repress various genes^29–31^.

Since we had previously identified three sgRNAs (CR-2A, CR-5A and CR-11) that were capable of binding to DUX4 exon 3 (Fig. 4b; top panel), we used these sgRNAs cloned in a vector that expresses just these sgRNAs and co-transfected them individually or as a pool with another vector expressing dCas9-KRAB along with a blue fluorescent protein (BFP) tag (See “Materials and methods” section) in DKO cells. *DUX4-fl* transcript levels were lowered in cells transfected with individual sgRNAs as well as pools, but the reduction was only found to be significant with CR-5A (Fig. 4b; graph). This could be attributed to low transfection efficiency and assessment of pooled DKO cells instead of single-cell clones. Nevertheless, we subsequently focused on this sgRNA (CR-5A) to repress DUX4 in patient myoblasts but BFP expression was undetectable by FACS, which was a hindrance in subsequent identification of targeted cells. Hence, we cloned CR-5A in a lentiviral vector that expresses both the sgRNA and a GFP-tagged dCas9-KRAB fusion protein from the same construct (See “Materials and methods” section). Transduction of this vector in patient myoblasts, followed by qRT-PCR analysis revealed that *DUX4-fl* is significantly downregulated in targeted myoblasts (Fig. 4c). We also assessed expression of target genes *TRIM43, MBD3L2* and *ZSCAN4* (Fig. 4d) and found that all three genes were downregulated in targeted cells to various extents, in comparison to controls. Out of these, the downregulation of *TRIM43* and *ZSCAN4* was statistically significant, while that of *MBD3L2* was not, and its reduction in transcript levels was much less pronounced compared to the other two genes.

To determine if DUX4 and target gene downregulation was due to bonafide increase in heterochromatin around the locus, we assessed levels of H3K9me3 and RNA polymerase II C-terminal domain phosphorylated at the Serine 2 residue (RNAPolII-S2) by quantitative ChIP (qChIP) using qPCR primers designed to the DUX4 promoter. As expected, we observed significant enrichment of H3K9me3 in targeted cells (Fig. 4e; left). We also observed a significant enrichment of RNAPolII-S2 at this locus (Fig. 4e; right) in targeted cells, compared to controls, likely reflecting the.

abundance of paused RNAPolII at the now heterochromatin (H3K9me3) rich locus.

## Discussion

We used two independent targeting strategies based on genome and epigenome editing, with a common end goal of repressing pathogenic DUX4, the gene that is central to FSHD pathology. The non-canonical polyadenylation sequence in the pLAM region (exon 3) of *DUX4* is the result of a critical single nucleotide polymorphism (SNP) that determines pathogenicity of a permissive 4qA allele^5^, and has long been associated with FSHD, likely through the stabilization of the resulting *DUX4-fl* mRNA^17^. In our first approach, using a pair of flanking CRISPR-Cas9 sgRNAs, we successfully deleted this poly-A sequence, in both HCT116 cells and in a patient myoblast cell line. In a previous study^5^, the authors used expression constructs harboring alternative poly-A sequences to show its importance in *DUX4-fl* expression. Additionally, distal *DUX4* elements have been targeted previously using antisense oligonucleotides^32^. However, to our knowledge, this is the first known report of successful permanent deletion of the endogenous pathogenic poly-A sequence of *DUX4*. Global genomic CpG demethylation by 5-Aza-C treatment, which can upregulate *DUX4-fl* expression in parental HCT116 cells, was unable to do so in a poly-A deleted single-cell clone (L24) that likely had both 4qA alleles targeted.

Conversely, independent single-cell clones that had only one poly-A targeted (M5 and DM2) did not show this effect, which strongly hints that this poly-A sequence is crucial for DUX4 mRNA stabilization as has been speculated before^5,17^. However, further assessment of nascent RNA levels needs to be performed to confirm this. After demonstrating the feasibility of targeting this locus, we extended this targeting strategy to an hTERT-immortalized myoblast cell line from an FSHD patient, in order to assess the downstream effects of poly-A deletion. We confirmed the presence of deletion of the poly-A in pooled FSHD myoblasts using two different sgRNA pairs. More importantly, in targeted patient myoblasts, we observed downregulation of not only D4Z4 transcript levels, but also a subset of *DUX4* target genes (*TRIM43* and *ZSCAN4*) that are robust biomarkers of the FSHD, validating the significance of the poly-A sequence in *DUX4* expression and FSHD pathogenesis. Intriguingly, in myoblasts, *MBD3L2,* another known marker for FSHD, did not show robust downregulation through DUX4 silencing by either of the two approaches. It is possible that there are indirect mechanisms of MBD3L2 expression that are independent of DUX4-dependent activation. Alternatively, this could be an idiosyncrasy of the cell line used in this study. Assessing the expression of this gene post-targeting of DUX4 in multiple independent FSHD cell lines may shed light on this.

Since DUX4 is epigenetically repressed in normal somatic tissues through increased CpG and repressive histone methylation at D4Z4, in our second approach for repressing *DUX4-fl*, we sought to increase levels of the heterochromatic marker H3K9me3 at this locus, using the dCas9-KRAB system, initially in pooled DKO cells and then, in patient myoblasts. Similar to the poly-A deletion, we showed that appropriate sgRNA/dCas9-KRAB combination significantly repressed *DUX4-fl* and target gene expression. Additionally, we confirmed that this repression was indeed due to H3K9me3 enrichment and spreading at D4Z4. Recently, another group has reported a similar study wherein they targeted the entire D4Z4 array, along with flanking upstream and downstream regions, with multiple sgRNAs and dCas9-KRAB^33^. The authors of this study showed that *DUX4-fl* and its target genes are downregulated by using different combination of sgRNAs across the D4Z4 array. However, the most potent sgRNAs in their study were in the DUX4 promoter and DUX4 open reading frame (exon 1). Although they had sgRNAs designed to exon 3, these did not repress *DUX4-fl* or its target genes. One of their sgRNAs (#10) overlaps with an sgRNA (CR-5; 20 bases) within exon 3, we had initially tested. They did not experience efficient binding with this sgRNA within exon 3, and similarly, we did not see any evidence of indels in our surveyor assay with this CR-5 (Fig. 1b; top panel). However, we did see both indels and an effect on heterochromatin enrichment when we used the truncated version of this sgRNA (CR-5A; 17 bases). This supports previous findings that usage of truncated sgRNAs (tru-sgRNAs) can improve specificity of targeting^34^, with reduced off-targeting effects and also reinforces the findings of this previous study. Interestingly, both CR-5 and CR-5A share a 12 bp seed sequence and a PAM (+ NGG) with the DUX4 promoter and another sgRNA (# 7) in the study by Himeda et al*.* found to effectively induce heterochromatin at the DUX4 promoter region. It is possible that the high levels of H3K9me3 observed at a region (DUX4 promoter) that is distant (> 1 kb) from the sgRNA binding site (exon 3) could be due to recruitment of dCas9-KRAB to the DUX4 promoter itself by sgRNA-5A. This is however unlikely, given the notion that only 1 bp mismatch (2 bp in some cases) at the 5’ end of sgRNAs is tolerated for sgRNA binding and activity^35^, and CR-5A has a 5 bp mismatch at the 5’ end. Alternatively, it is indicative of seeding of heterochromatin at exon 3, followed by spreading of repression throughout the D4Z4 array and the DUX4 promoter. In addition to elevated H3K9me3 levels, we observed enrichment of RNA PolII phosphorylated at serine 2, which is associated with transcriptional elongation^36^. Although this observation may seem ostensibly unexpected, it is possible that H3K9me3 enrichment pauses or decreases the movement of RNAPolII at this locus, which would be consistent with the currently held view of epigenetic modifications influencing the localization and activity of transcription^37^. This could be further verified by performing qChIP for PolII in the vicinity of the pLAM region in Exon 3. Also, it is important to note that since the primers for qChIP are not specific to the pathogenic 4q allele, they are detecting H3K9me3 and RNAPolII-S2 across D4Z4 alleles on all four chromosomes (4q and 10q). Intriguingly, the level of H3K9me3 detected in treated cells is almost twice that of the non-treated cells. This suggests extensive and long-range gain of H3K9me3 given that we assessed the promoter region located in each repeat, despite seeding heterochromatin from the unique Exon-3 region. The substantial gain in H3K9me3 may also reflect appreciable gains of H3K9me3 at both 4q alleles as well as 10q because the 5A gRNA has a single mismatch with 10q. Moreover, recent reports suggest that dCas9 directed in the vicinity of transcriptional start sites (TSSs) can physically block RNA polymerase passage^38^, which would also be consistent with our observations.

In this study, we assessed changes in mRNA expression of *DUX4*. DUX4 protein detection in myoblasts and myotubes is difficult, given the extremely stochastic nature and low frequency of expression (~ 1 in 1000 myonuclei in patient myotubes). In an earlier study^19^, we did not see conclusive results with commercially available DUX4 antibodies, possibly due to cross-reactivity to the related but non-pathogenic gene, DUX4c^39^. Nevertheless, the fact that target genes of DUX4 are repressed, supports the idea that DUX4 protein might be reduced in targeted cells. *DUX4-fl* repression seems to be a direct consequence of poly-A deletion, and not of lentiviral infection, since the same has been observed in HCT116 cells where lipofection and not transduction, was used to target cells. Additionally, appropriate negative controls that do not target *DUX4* and do repress target gene expression, rule out the possibility that target gene repression may be an artifact of lentiviral transduction itself. Interestingly, *DUX4-fl* downregulation observed was significantly higher in 5-Aza-C treated L24, compared to myoblasts. This likely reflects the fact that the former is a single-cell clone whereas we worked with pooled myoblasts. Single-cell cloning of targeted patient myoblasts, followed by differentiation to myotubes and western blotting for target gene expression will offer a more robust insight into long-term consequences of such targeting. It is important to note that here, we have tested only a small subset of DUX4 target genes of, as proof-of-concept of such an approach. DUX4 is a master transcriptional regulator that affects the expression of multiple genes during normal development, which are dysregulated in FSHD. Thus, it is extremely crucial that stringent and systematic analyses be carried out to verify if dysregulation of most (if not all) of these target genes are alleviated by these approaches.

In this study, we offer proof-of-concept of targeting the polyadenylation signal within exon 3 of pathogenic *DUX4.* However, there are a number of caveats that need to be kept in mind while interpreting the results. Although the four sgRNAs used in this study (CR-2A, CR-92U, CR-5A and CR-11) have a total of 2 potential off-targets that are pseudogenes (see Supplementary Table S4 online). The effect of targeting these pseudogenes on cellular phenotype also need to be assessed carefully. In our experiments, one pair of sgRNAs (2A + 11) showed significant downregulation of DUX4 and its targets, whereas another pair (92U + 11) showed variable effects on different genes, suggesting indirect effects could also be a concern. Although sequence alignment algorithms do not predict a huge difference between the two, it is also possible that mismatches are better tolerated in CR-92U and hence it cuts at multiple D4Z4 arrays (4q and 10q) whereas CR-2A may be limited to just 4q. Moreover, our study was carried out in a single myoblast cell line. For evaluating the generalizability of such approaches, it is crucial to carry out similar experiments in multiple, independent FSHD myoblasts. Moreover, *DUX4-fl* expression is low-abundant and heterogenous within myoblasts but is more robust when myoblasts differentiate into myotubes. It remains to be seen how targeting the polyadenylation signal affects *DUX4-fl* and target gene expression in myotubes. Nevertheless, since DUX4 mRNA, once aberrantly expressed in a small number of myonuclei within myotubes, can diffuse into surrounding myonuclei upon translation, facilitating the spread of FSHD-specific gene expression cascade across myotubes^40^, it seems logical to reason that destabilizing the poly-A at the myoblast stage might be more beneficial in the context of possible therapeutic approaches that could potentially build upon such targeting strategies in future.

## Materials and methods

### Oligonucleotides

All oligonucleotides were synthesized using the service of Eurofins Genomics. Oligonucleotide sequences are listed in Supplementary Table S1 (PCR primers) and Supplementary Table S5 (CRISPR sgRNAs). Scrambled sgRNA sequences were obtained from Origene.

### Cell lines and culture media

HCT116 (ATCC; No. CCL-247) male colon carcinoma epithelial cell line, and 293 T human embryonic kidney cell line (ATCC; No. CRL-1573) were obtained from the American Type Culture Collection (www.atcc.org). DKO was obtained from Dr. Bert Vogelstein’s laboratory at Johns Hopkins University School of Medicine. 12A-DEL and 12U-DEL are hTERT-immortalized myoblast cell lines obtained from deltoid muscles of a female FHSD1 patient (age 22yrs; 18 kb EcoRI/BlnI digests on shortened 4qA; FSHD D4Z4 digests are typically < 35 kb) and her unaffected female sibling (age 24yrs; 112 kb EcoRI/BlnI digests on both 4q alleles), respectively. Both myoblast cell lines were obtained from the Wellstone Center for FSHD research at University of Massachusetts Medical School, and have been previously characterized^41^. HCT116, DKO and 293 T cells were cultured in DMEM/High Glucose media (Caisson labs) according to manufacturer’s instructions, supplemented with 10% Fetal Bovine Serum (FBS; Gibco); 1% non-essential amino acids (Gibco); 1% penicillin–streptomycin-glutamine (PSG; Gibco). Culture media (LHCN) for myoblast proliferation consisted of four parts DMEM/High Glucose to one-part Medium 199 (Sigma) supplemented with 15% FBS, 20 mm HEPES (Sigma) and 2.5 ng/ml Human Hepatocyte Growth Factor (hHGF; EMD Millipore), supplemented with 0.055 µg/ml dexamethasone (Sigma), 0.03ug/ml zinc sulfate (EMD Millipore), 1.4ug/ml vitamin B12 (Sigma), 0.88 mM sodium pyruvate (Gibco) and 1% PSG. Cells were plated on dishes coated with 0.1% pigskin gelatin (Sigma). Cells were split at around 70% confluence to avoid differentiation and media was changed every 2–3 days.

### sgRNA design for poly-A deletion and H3K9me3 upregulation

Using publicly available genome sequence data (UCSC Genome browser; https://genome.ucsc.edu/)^42^ and sequence alignment software Sequencher (Gene Codes Corporation), we looked for sgRNAs spanning the length of DUX4 exon 3 (pLAM) and approximately 250 bp immediately downstream for poly-A deletion and 500 bp upstream of the DUX4 TSS for H3K9me3 upregulation at D4Z4. These sgRNAs were identified using already established criteria to maximize targeting success^26^ and searching for PAM sequence (NGG) recognized *by S. pyogenes* Cas9 protein. Additionally, we also identified sgRNAs using the Optimized CRISPR Design tool (http://crispr.mit.edu)^43^. Off-targets were identified using the online tool (http://www.rgenome.net/cas-offinder/new)^44^. Since less than 5% of mismatches of 2 or more bases are tolerated by sgRNAs^35^, we only considered those off-targets that had up to 1 mismatched base (see Supplementary Table S4 online).

### sgRNA cloning and expression plasmids

pSpCas9(BB)-2A-Puro (PX459; Catalog # 48139), pSpCas9(BB)-2A-GFP (PX458; Catalog # 48318), lentiGuide-Puro (Catalog # 52963), pL-CRISPR.EFS.tRFP (Catalog # 57819), pLKO5.sgRNA.EFS.GFP (Catalog # 57822), pLV hU6-sgRNA hUbC-dCas9-KRAB-T2a-GFP (Catalog # 71237), pHR-SFFV-dCas9-BFP-KRAB (Catalog # 46911), pMD2.G (Catalog # 12259) and psPAX2 (Catalog # 12260) were obtained from Addgene. sgRNAs were cloned in appropriate vectors according to manufacturer instructions. Top and bottom oligonucleotides constituting sgRNAs were cloned into appropriate plasmids optimized for mammalian expression. Cloned plasmids were subsequently sequenced for verification of correct sgRNA sequence and orientation.

### Surveyor assay for sgRNA transfected cells

For initial assessment of the nuclease and binding activity of Cas9 and sgRNAs, HCT116 or DKO cells were seeded at density of 2 × 10^5^ cells and transfected with 2500 ng of the appropriate sgRNA cloned in PX459 or PX458 vectors in HCT116 or DKO cells (or equal volume of water for the mock transfections) using Opti-MEM (Life Technologies) and Lipofectamine 3000 (Life Technologies). 72 h post-transfection, cells were harvested, and genomic DNA was isolated using the NucleoSpin Tissue Kit (Machery-Nagel). Efficiency of binding and cutting was then assessed using the Surveyor Mutation Detection Kit (Transgenomic Inc.) with primers DUX4-pA-CR-Surv-F1 and DUX4-pA-CR-Surv-R5 and HotStar Taq Plus (Qiagen) using thermocycling conditions described previously^23^. Final amplified products were examined on 3% agarose gel (Apex BioResearch Products) in 1X TBE.

### Deletion assay PCR with dual sgRNA system for DUX4 exon 3 poly-A deletion

PCR was performed with genomic DNA isolated from HCT116 or DKO cells using the Nucleospin Tissue kit (Machery Nagel) that were transiently transfected or transduced with sgRNA combinations flanking the DUX4 exon 3 poly-A (CR-2A and CR-11 or CR-92U and CR-11), cloned in suitable vectors or empty vectors (mock). PCR was carried out with HotStar Taq (Qiagen) using the following thermocycling conditions: initial denaturation of 5 min at 94 °C, followed by 40 cycles of 30 s at 94 °C, 30 s at 58 °C, 30 s at 72 °C along with a final extension step of 10 min at 72 °C. Amplified products were examined on 1.5% agarose (Agarose Unlimited) gels in 1X TAE. Primers used for detection of undeleted and deleted products were DUX4-pA-CR-Surv-F1 and DUX4-pA-CR-Surv-R5 (HCT116 and DKO) or DUX4-pA-CR-Surv-R6 (myoblasts). In myoblasts targeted with either CR-2A-11 or CR-92U-11 combination, PCR products were TA cloned using the NEB PCR Cloning Kit (New England Biolabs) and sequenced using forward and reverse primers provided with the kit.

### Generation of DUX4 exon 3 poly-A knockouts of HCT116 and fluorescence activated cell sorting (FACS) analysis

Two sgRNAs (CR-2A and CR-11) flanking the DUX4 exon 3 poly-A were independently cloned into PX458. 1250 ng of each cloned vector (2500 ng total DNA) was then transiently transfected into 2 × 10^5^ HCT116 or DKO cells seeded in six-well plates using reduced serum media (Opti-MEM) and Lipofectamine 3000 (Life Technologies). 72 h post-transfection, cells were enriched for GFP-positive cells (pX458) using FACS into three fractions depending on the intensity of GFP fluorescence above background: low (L), medium (M) and high (H); see Supplementary Fig. S3 online). This was done because high levels of GFP expression is sometimes known to be toxic to cells^24,25^ and we wanted to ensure viability of targeted clones by sorting low and medium intensity cells as well. We observed PCR amplified products corresponding to successful deletion in ‘low’ and ‘medium’ sorted fractions. Cells from these fractions were then plated at low dilutions to ensure growth of single-cell clones. Although we could not discern a corresponding product for the ‘high’ fraction based on the gel electrophoresis results, we plated these cells as well to avoid missing any targeted clone. A part of these enriched cells was harvested for deletion assay PCR and the rest were plated at dilutions corresponding to 1 cell every 3 wells of a 96-well plate to facilitate isolation of single-cell clones. After 2–3 weeks, clones were expanded, and genomic DNA isolated from a fraction of these clonal cells using the NucleoSpin Tissue Kit (Machery-Nagel). Subsequently, the deletion assay was performed using these clonal cells to identify positively targeted clones. PCR products of positive clones were then sequenced using forward and reverse primers used in the deletion assay (DUX4-pA-CR-Surv-F1 + DUX4-pA-CR-Surv-R5) to identify the nature of deletion. Sequenced reads were analyzed using Sequencher software (Gene Codes Corporation). Percentage matches are listed in Table 1 (for HCT116 targeted single-cell clones) and Supplementary Table S3A and B (targeted myoblast TA clones) and correspond to the topmost values obtained upon alignment to the human reference genome using the BLAT tool on the UCSC Genome Browser (Human GRCh37/hg19 build).

### CRISPR-based lentiviral transduction of hTERT-immortalized FSHD myoblasts for poly-A deletion and H3K9me3 upregulation

All lentiviral procedures were carried out according to Biosafety Level 2 guidelines.

Generation of lentivirus: All lentiviral particles were generated using the second-generation packaging system. For each virus, 7.2 × 10^6^ 293FT cells were seeded in a 10 cm dish with suitable antibiotic-free media. 24 h later, scrambled (vector only, non-targeting control) or target sgRNA-cloned lentiviral vectors were transfected in these cells with Opti-MEM (Life Technologies) and Lipofectamine 3000 (Life Technologies). 1 pmol of each lentiviral expression plasmid was used per transfection, co-transfected with the packaging vector (psPAX2; 1 pmol) and envelope vector (pMD2.G; 0.2 pmol). 18 h post-transfection, media was replenished with 10 ml of fresh antibiotic-free media. Media was changed, and viral supernatants were harvested at 48 h, 72 h and 96 h post-transfection, pooled and stored at 4 °C. The total pooled supernatant was immediately subjected to concentration of virus by mixing with 30 ml of Lenti-X Concentrator (Takara Bio USA; Cat # 631231), incubation at 4 °C for 2 h and then centrifugation at 1500 g for 45 min at 4 °C. The supernatant was then discarded, and the pellet was dissolved in 300ul of PBS, and immediately used for subsequent transduction.

Transduction of myoblasts: 25,000 cells of 12A-DEL FSHD myoblasts were seeded in each well of a 6-well plate (for each transduction), prior to the day of the first infection. Cells were first treated with polybrene (8 µg/ml) and then appropriate volumes of lentivirus was added. For poly-A deletion, cells were infected with combination of CR-2A or CR-92U (cloned in pL-CRISPR.EFS.tRFP) with CR-11 (cloned in pLKO5.sgRNA.EFS.GFP). For H3K9me3 upregulation, CR-5A (pLV hU6-sgRNA hUbC-dCas9-KRAB-T2a-GFP) was used. Four rounds of ‘spinfection’ were carried out (each at 8-h intervals), with media being changed after each infection, as described previously^33,45^. 24 h post-final infection, cells were expanded onto 10 cm dishes and with antibiotic-containing LHCN media. Cells were allowed to recover and expand for 7 days post-final infection before assessing GFP/tRFP fluorescence with a Nikon eclipse Ti-E inverted fluorescence microscope (Nikon Instruments Inc.). This method gave transduction efficiencies of ~ 100% for GFP- and tRFP-containing vectors. After assessing fluorescence, cells were harvested for genomic DNA and RNA isolation for measuring *DUX4* and target gene expression in transduced myoblasts.

### 5-Aza-C treatment

Cells from parental HCT116 and the poly-A knockout clones L24, DM2 and M5 were seeded at a concentration of 0.5 × 10^6^ cells/ml and maintained in 5-Aza-C (Sigma; Cat No. A3656, dissolved in water) supplemented media at a final concentration of 0.05 μM. Media was changed every 24-h for 3 days. Cells were harvested 24 h after the last treatment for subsequent isolation of total RNA and assessment of *DUX4-fl* expression.

### Chromatin immunoprecipitation (ChIP) and quantitative PCR (qPCR)

ChIP was carried out using protocols as described previously^19^. Protein-bound DNA was immunoprecipitated (IP samples) with either rabbit polyclonal anti-H3K9me3 (Active Motif; Cat # 39161), rabbit monoclonal anti-phospho-Rpb1 CTD (Ser2) (Cell Signaling Technology, Cat # 13499) or anti-or rabbit serum (RS) as negative control, and assessed by qPCR with DUX4-Prom-F1 and DUX4-Prom-R1. Chromatin controls not treated with any antibody was labeled as Input sample. Enrichment of H3K9me3 or RNA PolII in qChIP results was calculated after adjusting for input dilution, RS background and subsequently expressing corrected IP values as percentage of input. Triplicates were used for each sample.

### Isolation of RNA and preparation of cDNA

Total RNA was isolated from cells using the NucleoSpin RNA II kit (Machery-Nagel). First-strand cDNA was prepared from equal amounts of starting RNA (1ug total RNA) with either oligo-dT primers (for *DUX4-fl* analysis) or random hexamers (for D4Z4 transcription and target gene analysis), with and without M-MuLV reverse transcriptase (RT) according to the manufacturer’s instructions (NEB). cDNAs prepared with and without RT were used as templates for both qualitative and quantitative PCR. cDNA was diluted to one-fifth of original concentration prior to qualitative RT-PCR and qRT-PCR.

### qRT-PCR for DUX4 and its target genes

*DUX4-fl* expression was assessed using oligo-dT primed cDNA as template, whereas general D4Z4 transcription and target gene expression was assessed using random hexamer primed cDNA as template. qRT-PCR for *DUX4-fl* transcripts was carried out using previously published^19^ primers DUX4-cDNA-F10 and DUX4-cDNA-R4 that amplified spliced and polyadenylated pathogenic DUX4 transcripts containing exons 2 and 3. D4Z4 transcription was assessed using exon 1 primers DUX4-UTR-Fwd and DUX4-A-Rev. Target genes TRIM43, MBD3L2 and ZSCAN4 expression was quantified using previously published primers (TRIM43-Fwd and TRIM43-Rev)^46^, (MBD3L2-Fwd and MBD3L2-Rev)^46^, and (ZSCAN4-F1 + ZSCAN4-R1)^18^, respectively. Expression for each sample was analyzed relative to GAPDH or ACTB, with GAPDH-q-Fwd and GAPDH-q-Rev (for HCT116 experiments and untransduced myoblasts) or ACTB-q-Fwd and ACTB-q-Rev (for lentivirally transduced myoblast experiments), using the ΔΔCt method. qRT-PCR was performed with the same SYBR Green mastermix, reagents and conditions as previously described^19^. Triplicates were used for each sample.

### Statistical analyses

#### H3K9me3 qChIP

Effective enrichment (IP–RS) values (normalized to and expressed as percentage of input) were used to determine statistically significant differences between pairs of samples using a paired two-tailed student’s t-test.

#### *DUX4-fl* and target gene qRT-PCR

Expression values were subsequently used to determine statistically significant differences by carrying out either multiple comparisons using one-way ANOVA (for datasets having more than two groups) or an unpaired two-tailed student’s t-test (for datasets comprising of only two samples) using GraphPad Prism 9.0. A complete list of statistical analyses and resulting p-values from all statistical analyses can be found in Supplementary Table S6 (ANOVA) and Supplementary Table S2 (t-test) online.

## Supplementary Information

Supplementary Information.

Supplementary Table S2.

Supplementary Table S6.
