# Supplementary material for: CRISPR mediated targeting of DUX4 distal regulatory element represses DUX4 target genes dysregulated in Facioscapulohumeral muscular dystrophy
Source: Sci Rep. 2021 Jun 15;11:12598. doi: 10.1038/s41598-021-92096-0 (PMC8206090; doi:10.1038/s41598-021-92096-0)
Supplement: Supplementary file 1 — Supplementary Information. [file 41598_2021_92096_MOESM1_ESM.docx]

**Supplementary Information**

**CRISPR mediated targeting of *DUX4* distal regulatory element represses DUX4 target genes dysregulated in Facioscapulohumeral muscular dystrophy**

**Sunny Das and Brian P. Chadwick**


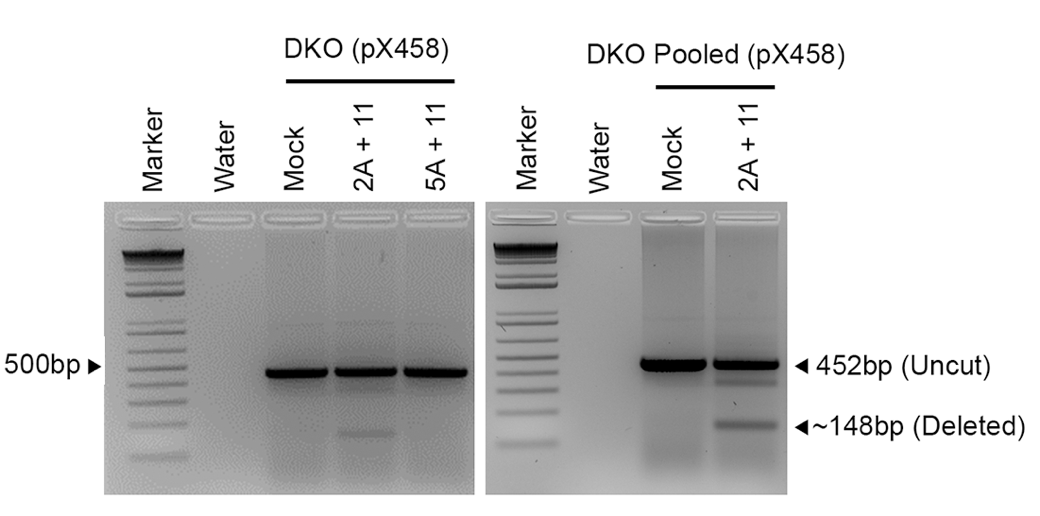


**Supplementary Figure S1.** Repetition and validation of the deletion assay with the CR-2A and CR-11 sgRNA combination prior to sorting (left gel) and in pooled cells post-sorting for GFP+ cells (right gel) in the DKO cell line. ‘Pooled’ refers to a heterogenous mixture of sorted cells, as opposed to single-cell clones that we were trying to generate from these targeted DKO cells. Sample names are indicated on top along with cell line used for transfection and vector in which sgRNAs were cloned. The expected size for undeleted and deleted PCR products is indicated on the right-hand side by black triangles.

**Supplementary Figure S2**. Sequencing reads from DUX4 exon 3 polyadenylation sequence targeted single-cell clones of HCT116. The sequence shown corresponds to 268,529 – 268,978 of Chr4_gI877872_fix hg19 UCSC Genome Browser. The blue boxed region shows sgRNA CR-2A whereas the red boxed region shows sgRNA CR-11. The poly-A signal is highlighted in black. The white bases on a grey background indicate the deleted region in each clone.

**Clone-DM2**

GGCGGTCAAAAGCATACCTCTGTCTGTCTTTGCCCGCTTCCTGGCTAGAC

CTGCGCGCAGTGCGCACCCCGGCTGACGTGCAAGGGAGCTCGCTGGCCTC

TCTGTGCCCTTGTTCTTCCGTGAAATTCTGGCTGAATGTCTCCCCCCACC

TTCCGACGCTGTCTAGGCAAACCTGGATTAGAGTTACATCTCCTGGATGA

TTAGTTCAGAGATATATTAAAATGCCCCCTCCCTGTGGATCCTATAGAAG

ATTTGCATCTTTTGTGTGATGAGTGCAGAGATATGTCACAATATCCCCTG

TAGAAAAAGCCTGAAATTGATTTACAGAACTTCGGTGATCAGTGCAGATG

TGTTTCAGAACTCCATAGTAGACTGAACCTAGAGAATGGTTACATCACTT

AGGTGATCAGTGTAGAGATATGTTAAAATTCTCGTGTAGACAGAGCCTAG

**Clone-L24**

GGCGGTCAAAAGCATACCTCTGTCTGTCTTTGCCCGCTTCCTGGCTAGAC

CTGCGCGCAGTGCGCACCCCGGCTGACGTGCAAGGGAGCTCGCTGGCCTC

TCTGTGCCCTTGTTCTTCCGTGAAATTCTGGCTGAATGTCTCCCCCCACC

TTCCGACGCTGTCTAGGCAAACCTGGATTAGAGTTACATCTCCTGGATGA

TTAGTTCAGAGATATATTAAAATGCCCCCTCCCTGTGGATCCTATAGAAG

ATTTGCATCTTTTGTGTGATGAGTGCAGAGATATGTCACAATATCCCCTG

TAGAAAAAGCCTGAAATTGATTTACAGAACTTCGGTGATCAGTGCAGATG

TGTTTCAGAACTCCATAGTAGACTGAACCTAGAGAATGGTTACATCACTT

AGGTGATCAGTGTAGAGATATGTTAAAATTCTCGTGTAGACAGAGCCTAG

**Clone-M5**

GGCGGTCAAAAGCATACCTCTGTCTGTCTTTGCCCGCTTCCTGGCTAGAC

CTGCGCGCAGTGCGCACCCCGGCTGACGTGCAAGGGAGCTCGCTGGCCTC

TCTGTGCCCTTGTTCTTCCGTGAAATTCTGGCTGAATGTCTCCCCCCACC

TTCCGACGCTGTCTAGGCAAACCTGGATTAGAGTTACATCTCCTGGATGA

TTAGTTCAGAGATATATTAAAATGCCCCCTCCCTGTGGATCCTATAGAAG

ATTTGCATCTTTTGTGTGATGAGTGCAGAGATATGTCACAATATCCCCTG

TAGAAAAAGCCTGAAATTGATTTACAGAACTTCGGTGATCAGTGCAGATG

TGTTTCAGAACTCCATAGTAGACTGAACCTAGAGAATGGTTACATCACTT

AGGTGATCAGTGTAGAGATATGTTAAAATTCTCGTGTAGACAGAGCCTAG





\

**Supplementary Figure S3.** Results from FACS analysis of HCT116 cells transfected with CR-2A and CR-11 gRNAs cloned in pX458 for subsequent single-cell cloning

**Supplementary Table S1.** List of PCR and sequencing primers.

| Category | Oligonucleotide | Sequence (5' to 3') | Product size (for PCR primers) |
| --- | --- | --- | --- |
| DUX4 poly-A CRISPR Surveyor assay/Deletion assay | DUX4-pA-CR-Surv-F1  DUX4-pA-CR-Surv-R5  DUX4-pA-CR-Surv-R6 | GGCGGTCAAAAGCATACCTC  GTCTAGGCTCTGTCTACACG  GAACACTTGCCTACACTCTG | 452bp  528bp |
| DUX4-fl qRT-PCR Exon 1 primers | DUX4-UTR-Fwd  DUX4-A-Rev | AGGCGCAACCTCTCCTAGAAAC  GCTCCTCCAGCAGAGCCCGGTATTC | 114bp |
| DUX4-fl qRT-PCR  Exon 2-3 primers | DUX4-cDNA-F10  DUX4-cDNA-R4 | ACCGCGGAGAACTGCCATTC  GACATTCAGCCAGAATTTCACG | 159bp |
| TRIM43 qRT-PCR | TRIM43-Fwd  TRIM43-Rev | ACCCATCACTGGACTGGTGT  CACATCCTCAAAGAGCCTGA | 100bp |
| MBD3L2 qRT-PCR | MBD3L2-Fwd  MBD3L2-Rev | CGTTCACCTCTTTTCCAAGC  AGTCTCATGGGGAGAGCAGA | 142bp |
| ZSCAN4 qRT-PCR | ZSCAN4-F1  ZSCAN4-R1 | TGGAAATCAAGTGGCAAAAA  CTGCATGTGGACGTGGAC | 93bp |
| GAPDH qRT-PCR | GAPDH-q-Fwd  GAPDH-q-Rev | CCCAATACGACCAAATCCGT  TCTCTGCTCCTCCTGTTCGA | 119bp |
| ACTB qRT-PCR | ACTB-q-Fwd  ACTB-q-Rev | CTCTTCCAGCCTTCCTTCCT  AGCACTGTGTTGGCGTACAG | 116bp |
| ChIP qPCR | DUX4-Prom-F1  DUX4-Prom-R1 | CCTGTTGCTCACGTCTCTCC  CACACTGCAGACTCCCCAC | 164bp |

**Supplementary Table S3**. Sequencing reads from representative DUX4 exon 3 polyadenylation sequence targeted TA clones from FSHD myoblasts using the **(A)** CR-2A + CR-11 or **(B)** CR-92U + CR-11 combination. Sequences with matches to chromosome 10 are not evaluated for indels.

**A) 2A + 11**

| **TA Clone** | **Best match** | **Description** |
| --- | --- | --- |
| C5 | 98.9% Chr4 | - No changes - Intact poly-A |
| C6 | 100% Chr4 | - Deletion of 204bp at CR-11 - Intact poly-A |
| C10 | 99.8% Chr10 | - |

**B) 92U + 11**

| **TA Clone** | **Best match** | **Description** |
| --- | --- | --- |
| C2 | 99.7% Chr4 | - 34bp deletion at CR-92U - 101bp deletion at CR-11 - Intact poly-A sequence |
| C3 | 99.7% Chr10 | - |
| C6 | 99.1% Chr4 | - 28bp deletion at CR-92U - Intact poly-A sequence |
| C8 | 99.1% Chr10 | - |
| C10 | 98.6% Chr4 | - 235bp deletion spanning poly-A |

**Supplementary Table S4.** Off-targets for sgRNAs used for targeting HCT116 cells and FSHD myoblasts. Results with 0 to 1 base mismatch are depicted; ‘+’ and ‘-‘ refer to sense and antisense strands, respectively. For a full list of mismatches, plug in sgRNA sequence from Table 2 into off-target finder tool (See Methods; <http://www.rgenome.net/cas-offinder/new>).

| sgRNA | Chromosome | Position | Direction | Mismatches | Gene | Function |
| --- | --- | --- | --- | --- | --- | --- |
| CR-2A | chr18 | 111748 | - | 0 | ROCKP1P | Pseudogene |
| CR-2A | chrY | 11308782 | + | 1 | DUX4L16 | Pseudogene |
| CR-2A | chr10 | 133685549 | + | 1 | None | NA |
| CR-2A | chr10 | 133761738 | + | 1 | None | NA |
| CR-5A | chr10 | 133685597 | + | 1 | None | NA |
| CR-5A | chr10 | 133761786 | + | 1 | None | NA |
| CR-92U | chr18 | 111665 | + | 0 | ROCKP1P | Pseudogene |
| CR-92U | chr10 | 133685631 | - | 1 | None | NA |
| CR-92U | chr10 | 133761820 | - | 1 | None | NA |
| CR-11 | chr10 | 133685853 | + | 0 | None | NA |
| CR-11 | chr10 | 133762042 | + | 0 | None | NA |

**Supplementary Table S5.** List of all CRISPR sgRNA adapter oligonucleotides.

| sgRNA oligonucleotide | Sequence (5' to 3') |
| --- | --- |
| DUX4-pA-CR1-T | CACCGCGCGCAGTGCGCACCC |
| DUX4-pA-CR1-B | AAACGGGTGCGCACTGCGCGC |
| DUX4-pA-CR2-T | CACCGCGCACCCCGGCTGACGTGCA |
| DUX4-pA-CR2-B | AAACTGCACGTCAGCCGGGGTGCGC |
| DUX4-pA-CR3-T | CACCGCACCCCGGCTGACGTGCAAG |
| DUX4-pA-CR3-B | AAACCTTGCACGTCAGCCGGGGTGC |
| DUX4-pA-CR4-T | CACCGACGTGCAAGGGAGCTCGC |
| DUX4-pA-CR4-B | AAACGCGAGCTCCCTTGCACGTC |
| DUX4-pA-CR5-T | CACCGCTTGTTCTTCCGTGAAATTC |
| DUX4-pA-CR5-B | AAACGAATTTCACGGAAGAACAAGC |
| DUX4-pA-CR5A-T | CACCGTTCTTCCGTGAAATTC |
| DUX4-pA-CR5A-B | AAACGAATTTCACGGAAGAAC |
| DUX4-pA-CR6-T | CACCGCCCACCTTCCGACGCTGTCT |
| DUX4-pA-CR6-B | AAACAGACAGCGTCGGAAGGTGGGC |
| DUX4-pA-CR7-T | CACCGACGCTGTCTAGGCAAACC |
| DUX4-pA-CR7-B | AAACGGTTTGCCTAGACAGCGTC |
| DUX4-pA-CR8-T | CACCGGATTAGAGTTACATCTCC |
| DUX4-pA-CR8-B | AAACGGAGATGTAACTCTAATCC |
| DUX4-pA-CR9-T | CACCGATTAAAATGCCCCCTCCCTG |
| DUX4-pA-CR9-B | AAACCAGGGAGGGGGCATTTTAATC |
| DUX4-pA-CR10-T | CACCGAAATTGATTTACAGAACTT |
| DUX4-pA-CR10-B | AAACAAGTTCTGTAAATCAATTTC |
| DUX4-pA-CR11-T | CACCGTAGACTGAACCTAGAGAA |
| DUX4-pA-CR11-B | AAACTTCTCTAGGTTCAGTCTAC |
| DUX4-pA-CR12-T | CACCGAGAATGGTTACATCACTT |
| DUX4-pA-CR12-B | AAACAAGTGATGTAACCATTCTC |
| DUX4-pA-CR2A-T | CACCGCACCCCGGCTGACGTGCA |
| DUX4-pA-CR2A-B | AAACTGCACGTCAGCCGGGGTGC |
| DUX4-pA-CR4A-T | CACCGTGCAAGGGAGCTCGC |
| DUX4-pA-CR4A-B | AAACGCGAGCTCCCTTGCAC |
| DUX4-pA-CR9A-T | CACCGAAAATGCCCCCTCCCTG |
| DUX4-pA-CR9A-B | AAACCAGGGAGGGGGCATTTTC |
| DUX4-pA-CR10A-T | CACCGTTGATTTACAGAACTT |
| DUX4-pA-CR10A-B | AAACAAGTTCTGTAAATCAAC |
| DUX4-pA-CR11A-T | CACCGACTGAACCTAGAGAA |
| DUX4-pA-CR11A-B | AAACTTCTCTAGGTTCAGTC |
| DUX4-pA-CR12A-T | CACCGAATGGTTACATCACTT |
| DUX4-pA-CR12A-B | AAACAAGTGATGTAACCATTC |
| DUX4-pA-92U-T | CACCGCCTAGACAGCGTCGGAAGG |
| DUX4-pA-92U-B | AAACCCTTCCGACGCTGTCTAGGC |
| Scrambled gRNA-T | CCACGCACTACCAGAGCTAACTCA |
| Scrambled gRNA-B | AAACTGAGTTAGCTCTGGTAGTGC |
